# Dialysis-associated steal syndrome as a rare complication of arteriovenous fistula angioplasty

**DOI:** 10.1093/jscr/rjae645

**Published:** 2024-10-10

**Authors:** Fang Nian Joanne Lim, Zhi Peng Nick Ng

**Affiliations:** Yong Loo Lin School of Medicine, National University of Singapore, 10 Medical Drive, Singapore 117597, Singapore; Department of Vascular Surgery, Singapore General Hospital, Outram Road, Singapore 169608, Singapore

**Keywords:** steal syndrome, arteriovenous fistula angioplasty, revision using distal inflow

## Abstract

The angioplasty of arteriovenous fistulas (AVF) has become indispensable in preserving haemodialysis access. Though well-known complications, such as pseudoaneurysm formation and rupture, can occur, the incidence of severe dialysis-associated steal syndrome (DASS) following AVF angioplasty is low. We describe a patient with limited dialysis options who developed significant DASS following angioplasty of his AVF. After excluding flow-limiting causes of DASS, the patient underwent a successful Revision Using Distal Inflow procedure, redirecting blood flow to his distal arm. Subsequently, all ischaemic symptoms resolved. While effective surgical options are available for the management of DASS, preventive measures such as proper angioplasty balloon sizing remain key.

## Introduction

Angioplasty of arteriovenous fistulas (AVF) is essential in maintaining the longevity of haemodialysis access. While oversizing angioplasty balloons can cause rupture and pseudoaneurysm formation, reports of clinically significant dialysis-associated steal syndrome (DASS) following AVF angioplasty remain scarce in the current literature.

DASS is an infrequent but disabling complication occurring in 1%–8% of patients with arteriovenous access [1]. Caused by a differential resistance between two vascular beds supplied by a single arterial inflow, blood flow is preferentially directed towards the low-resistance bed (the AVF) and away from the high-resistance bed (the distal extremity). This causes symptoms ranging from numbness and pain to major tissue loss.

We present a case of a patient who developed significant DASS after AVF angioplasty.

## Case report

**Figure 1:**
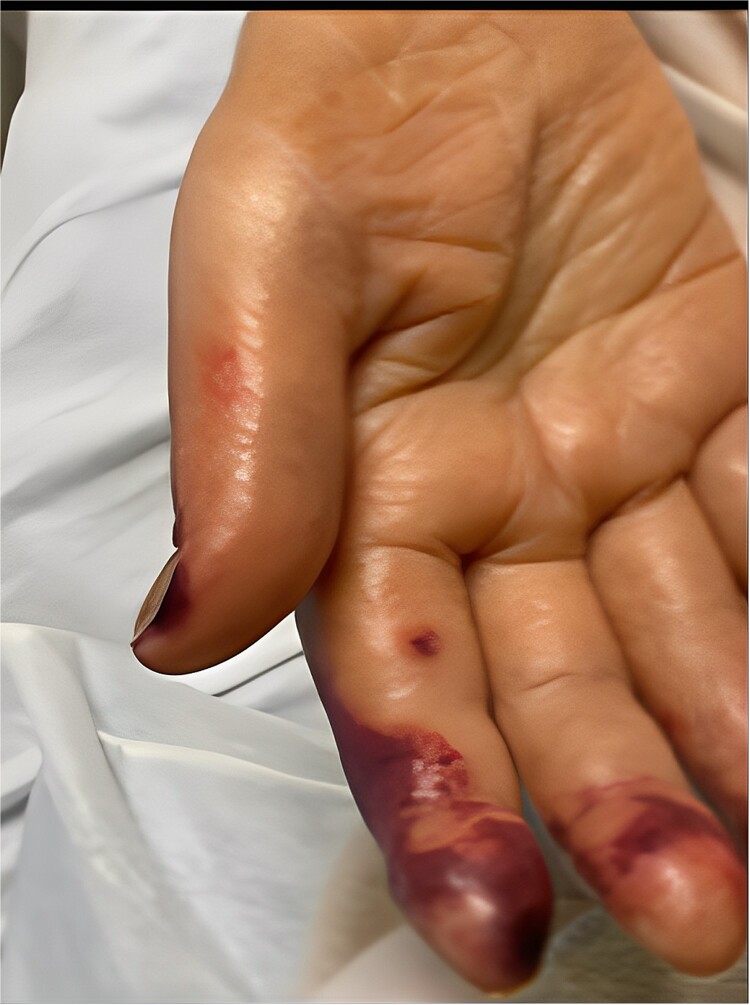
Digital ischaemia on the right hand.

A 58-year-old smoker with a history of type II diabetes, ischemic heart disease, and end-stage renal disease developed intense pain, discoloration, and blistering of his right thumb and fingertips 2 days after angioplasty of his 8-month-old right brachiocephalic AVF (Fig. 1).

**Figure 2:**
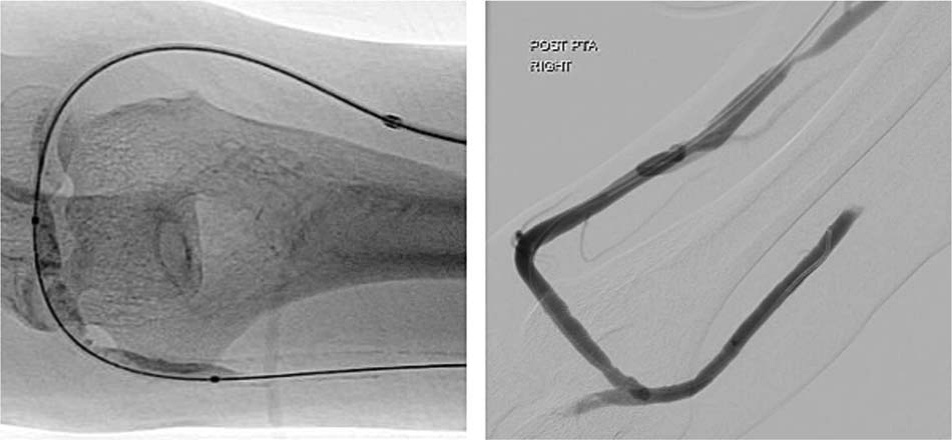
Angioplasty of the arteriovenous fistula 2 days prior to clinical presentation.

During the procedure, juxta-anastomotic and anastomotic stenoses of up to 75% were treated using non-compliant Mustang™ (Boston Scientific) balloon catheters (5 × 40 mm and 6 × 40 mm), inflated serially at 20 atm for 2 minutes each (Fig. 2). There was an improved palpable thrill after the angioplasty.

**Figure 3:**
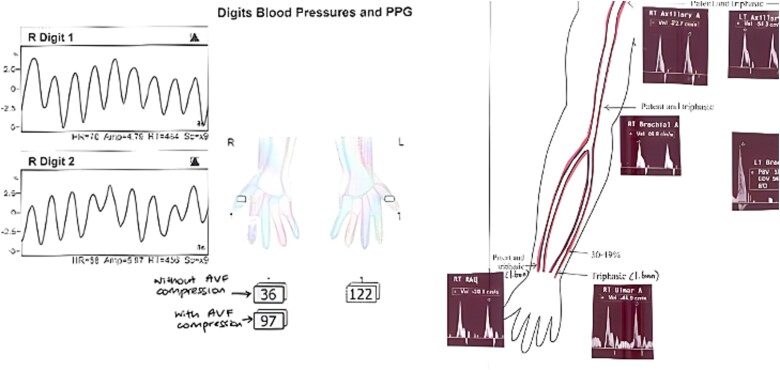
Investigations confirming dialysis-associated steal syndrome (DASS).

Upon examination 2 days later, the patient’s right hand was significantly colder than his left, with a prolonged capillary refill time of 5 seconds. Pain was alleviated and Doppler signals of his distal arteries were audible only with AVF compression. His digital finger pressure was 36 mmHg, rising to 97 mmHg with fistula compression. A duplex ultrasound revealed patent brachial, ulnar and radial arteries, confirming the diagnosis of DASS (Fig. 3).

While ligation of the AVF was considered, the patient’s long-term dialysis access options were severely limited. His history of bilateral lower limb deep venous thrombosis and a laparotomy for perforated sigmoid diverticulitis also rendered him incompatible for a thigh access graft or peritoneal dialysis.

Veins of both his forearms were unsuitable for new access creation due to thrombosis from multiple phlebotomies and cannulations. Additionally, his left brachial artery had a 75% stenosis with occlusion of the distal radial artery, increasing the risk of left-sided steal syndrome if a graft was to be placed.

Prior to creation of the AVF, the patient had multiple emergent admissions for fluid overload requiring urgent tunnelled vascular catheter insertion for dialysis. This was complicated with catheter-related bloodstream infections exacerbated by immunosuppression from retroviral therapy, making placement of a dialysis access catheter undesirable as a treatment option.

Fortunately, a short superficial cephalic tributary vein which could be harvested was identified on his right forearm. The decision was thus made for the patient to undergo a Revision Using Distal Inflow (RUDI) procedure to redirect flow from the fistula into the distal arm.

**Figure 4:**
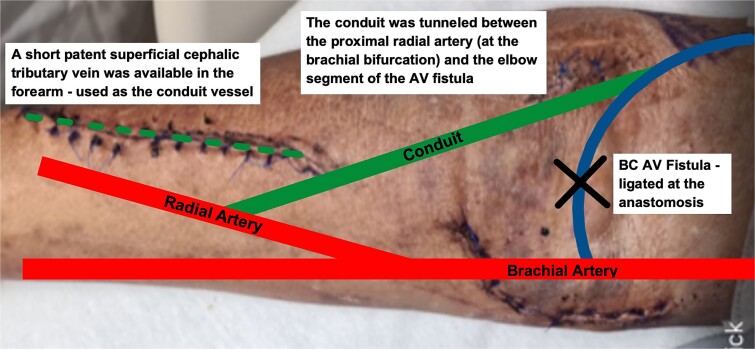
Schematic representation of RUDI. procedure

The harvested vein was tunnelled between the proximal radial artery (at the brachial bifurcation) and the elbow segment of the AVF. The AVF was then ligated at the anastomosis. A thrill was well felt in the upper arm after the procedure (Fig. 4).

**Figure 5:**
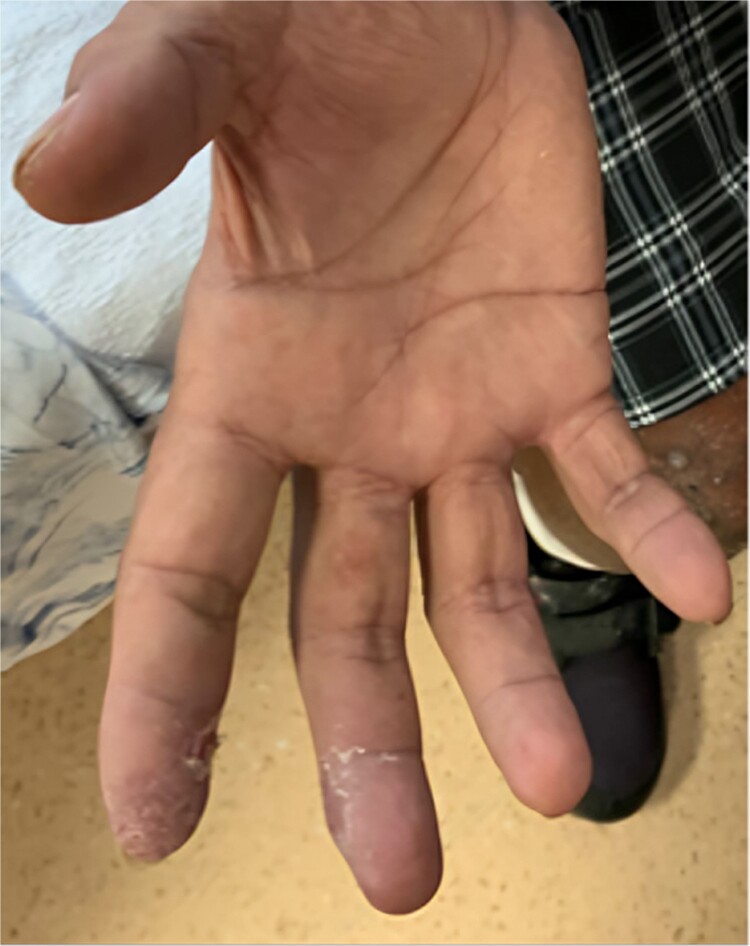
Resolution of ischaemic symptoms after the RUDI procedure.

**Figure 6:**
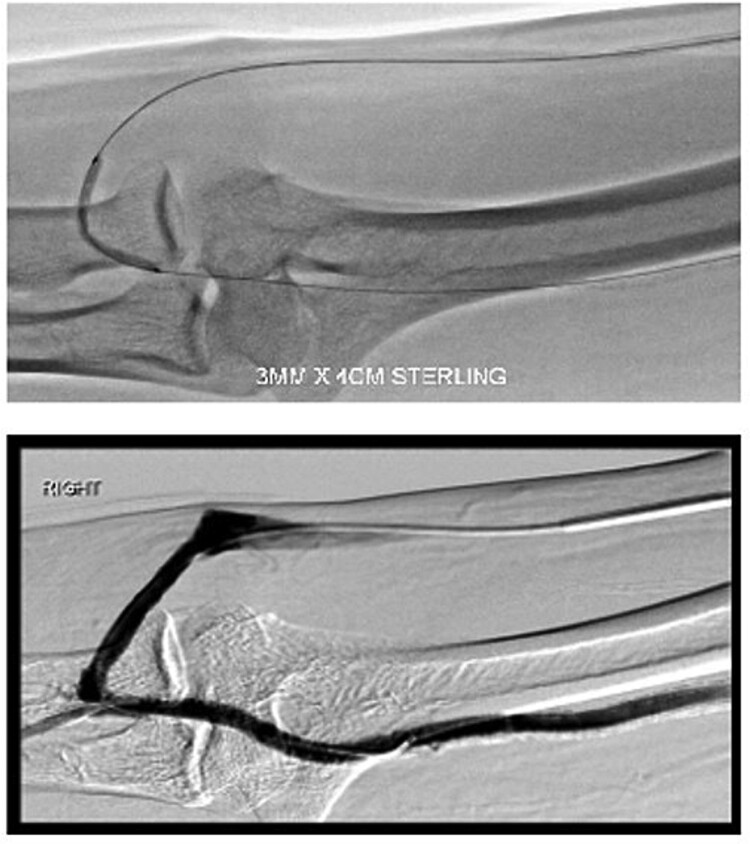
Angioplasty of the RUDI conduit vessel.

Post-operatively, all symptoms of ischaemia resolved (Fig. 5). The patient’s pulses were restored and dialysis resumed without complications. A gentle angioplasty of the conduit vessel was performed 1 month later to maintain access flow (Fig. 6). The access site remained functional at the 12-month follow-up.

## Discussion

Commonly recognised as a complication of arteriovenous access creation rather than AVF angioplasty [1], clinically significant DASS can result in drastic neurologic injury and tissue loss [2].

A comprehensive evaluation of all flow-limiting causes of DASS is imperative prior to AVF revision. In a case reported by Zamani *et al*. [3], an undetected subclavian artery stenosis resulted in angioplasty increasing fistula flow and exacerbating DASS to cause digital ischaemia. The patient was successfully managed with endovascular stenting, preventing the need for surgical intervention, which should be executed only when proximal and distal arterial disease is absent.

While ligation is the simplest mode of treatment, it sacrifices the vascular access site. With more patients having limited dialysis access options, reverting to catheter-directed dialysis places them at high risk of frequent thrombosis and life-threatening septicaemia [4]. Ligation should therefore be reserved for patients with limited life expectancy, previous failed steal-correcting procedure, ischaemic monomelic neuropathy (a severe variant of DASS involving ischaemic nerve damage leading to sensorimotor dysfunction) or gross ischaemic lesions [5]. In most individuals, salvaging the access site becomes paramount.

RUDI is a well-established procedure for DASS management, reducing access flow by >50% [6]. Though typically used in high flow fistulas, our case demonstrates that even with flow reduction in a non-high flow access, dialysis can still be effectively performed and the affected tissue can recover afterwards.

Other surgical methods such as Distal Revascularization with Interval Ligation (DRIL) and Proximalization of Arterial Inflow (PAI) can be employed to correct DASS in patients without flow-limiting stenoses.

DRIL involves anastomosing a low-resistance conduit vessel from a site proximal to the original AVF to the distal outflow artery. The native artery is then ligated distally to the AVF to prevent retrograde flow [7]. Similar to RUDI, DRIL requires a suitable vein for harvest.

In PAI, the arterial supply of the AVF is brought proximally with a prosthetic graft [8]. This increases inflow supply and recruits collaterals to enhance perfusion to the distal limb. Unlike RUDI and DRIL, PAI does not require ligation of the native artery. However, using a prosthetic graft increases the risk of thrombosis and infection [5].

Though significant DASS as a complication of AVF angioplasty is rarely seen, it is something a junior interventionist should be mindful of when sizing angioplasty balloons. Special care should be taken in patients with increased risks, including females, those over 60 years of age, individuals with diabetes mellitus, previous operations on the same limb or use of the brachial artery as the access donor vessel [9].

## Conclusion

Clinically significant DASS following AVF angioplasty, though uncommon, can lead to severe complications. Whilst prevention with careful balloon sizing during angioplasty is best, procedures such as RUDI provide an effective solution.
